# Corrigendum: Germline-Encoded TCR-MHC Contacts Promote TCR V Gene Bias in Umbilical Cord Blood T Cell Repertoire

**DOI:** 10.3389/fimmu.2020.01752

**Published:** 2020-08-13

**Authors:** Kai Gao, Lingyan Chen, Yuanwei Zhang, Yi Zhao, Ziyun Wan, Jinghua Wu, Liya Lin, Yashu Kuang, Jinhua Lu, Xiuqing Zhang, Lei Tian, Xiao Liu, Xiu Qiu

**Affiliations:** ^1^BGI Education Center, University of Chinese Academy of Sciences, Shenzhen, China; ^2^BGI-Shenzhen, Shenzhen, China; ^3^Division of Birth Cohort Study, Guangzhou Women and Children's Medical Center, Guangzhou Medical University, Guangzhou, China; ^4^School of Biology and Biological Engineering, South China University of Technology, Guangzhou, China; ^5^Department of Women and Children's Health Care, Guangzhou Women and Children's Medical Center, Guangzhou Medical University, Guangzhou, China; ^6^Department of Obstetrics and Gynecology, Guangzhou Women and Children's Medical Center, Guangzhou Medical University, Guangzhou, China

**Keywords:** TRBV gene usage, MHC genetic variations, quantitative trait locus mapping, umbilical cord blood, TCR-MHC co-evolution

In the original article, there was an error in the **Data Availability** statement. The original statement is as follows:

“The datasets that support the findings of this study are deposited under supervision and control in the China National Genebank (CNGB, https://db.cngb.org/cnsa/). Data of this project could be accessed after an approval application. Please refer to https://db.cngb.org/, or email: CNGBdb@cngb.org for detailed application guidance. The accession code CNP0000425 should be included in the application.”

The corrected statement is as follows:

“According to national legislation/guidelines, specifically the Administrative Regulations of the People's Republic of China on Human Genetic Resources (http://www.gov.cn/zhengce/content/2019-06/10/content_5398829.htm, http://english.www.gov.cn/policies/latest_releases/2019/06/10/content_281476708945462.htm), no additional data are available at this time. Data of this project can be accessed after an approved application to the Ministry of Science and Technology of the People's Republic of China (http://www.most.cn/bszn/new/rlyc/fwzn/). Please refer to https://fuwu.most.gov.cn/html/bszx/xzxkl/20190630/2907.html for detailed application guidance. The accession code CNSA-CNP0000425 should be included in the application.”

The authors apologize for this error and state that the revision does not change the scientific conclusions of the article in any way. The original article has been updated.

