# Supplementary material for: Germline-Encoded TCR-MHC Contacts Promote TCR V Gene Bias in Umbilical Cord Blood T Cell Repertoire
Source: Front Immunol. 2019 Aug 30;10:2064. doi: 10.3389/fimmu.2019.02064 (PMC6730489; doi:10.3389/fimmu.2019.02064)
Supplement: Supplementary file 11 [file Image_1.pdf]

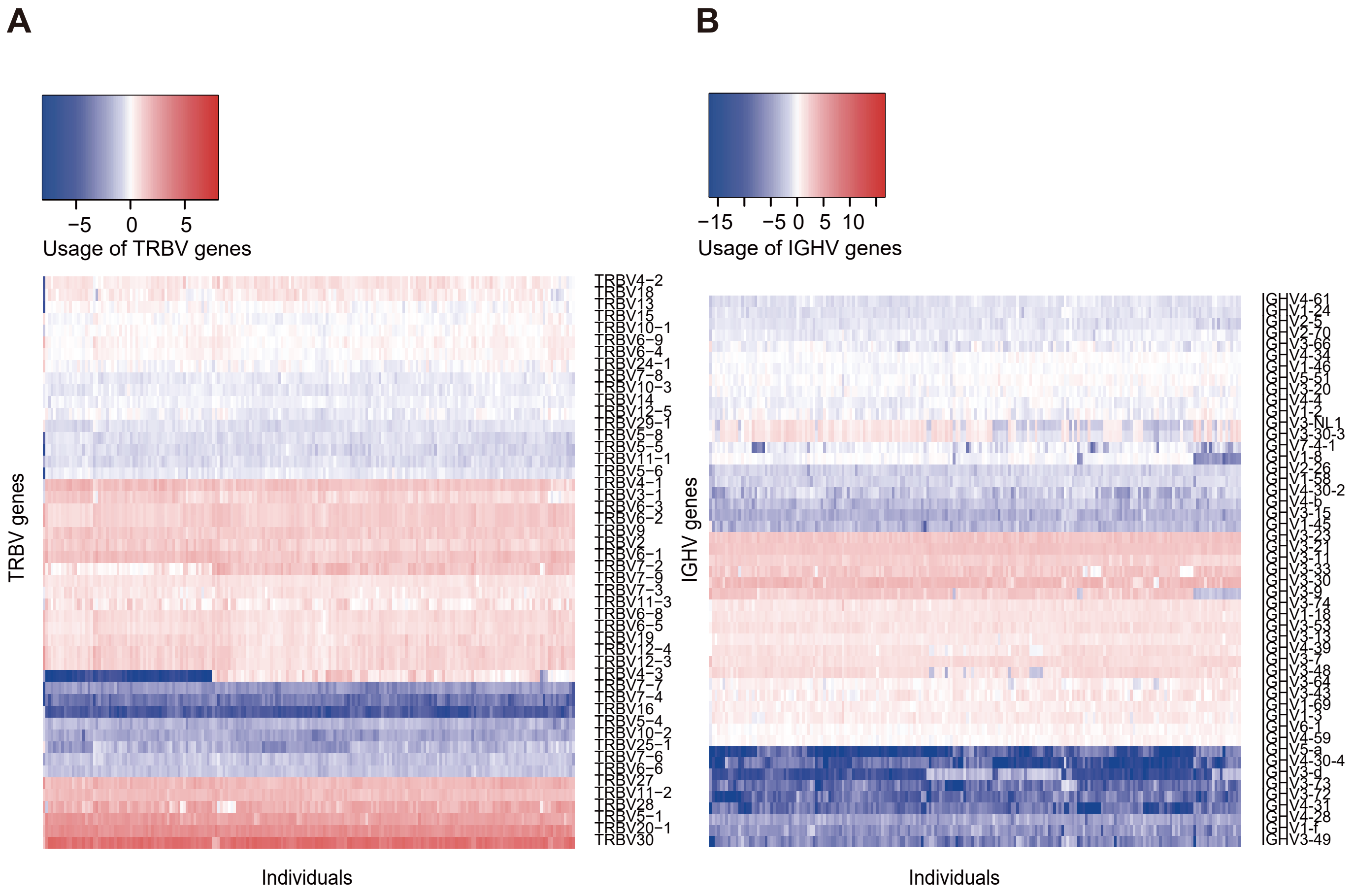

Supplementary Figure 1. The frequencies of TRBV and IGHV genes. Log2-transformed frequencies of TRBV **(A)** and IGHV **(B)**. 0.01 and 0.00001 pseudo-usage was added to avoid zeroes for TRBV and IGHV, respectively. Rows and columns were clustered using hierarchical clustering.
