# Supplementary material for: Germline-Encoded TCR-MHC Contacts Promote TCR V Gene Bias in Umbilical Cord Blood T Cell Repertoire
Source: Front Immunol. 2019 Aug 30;10:2064. doi: 10.3389/fimmu.2019.02064 (PMC6730489; doi:10.3389/fimmu.2019.02064)
Supplement: Supplementary file 12 [file Image_2.pdf]

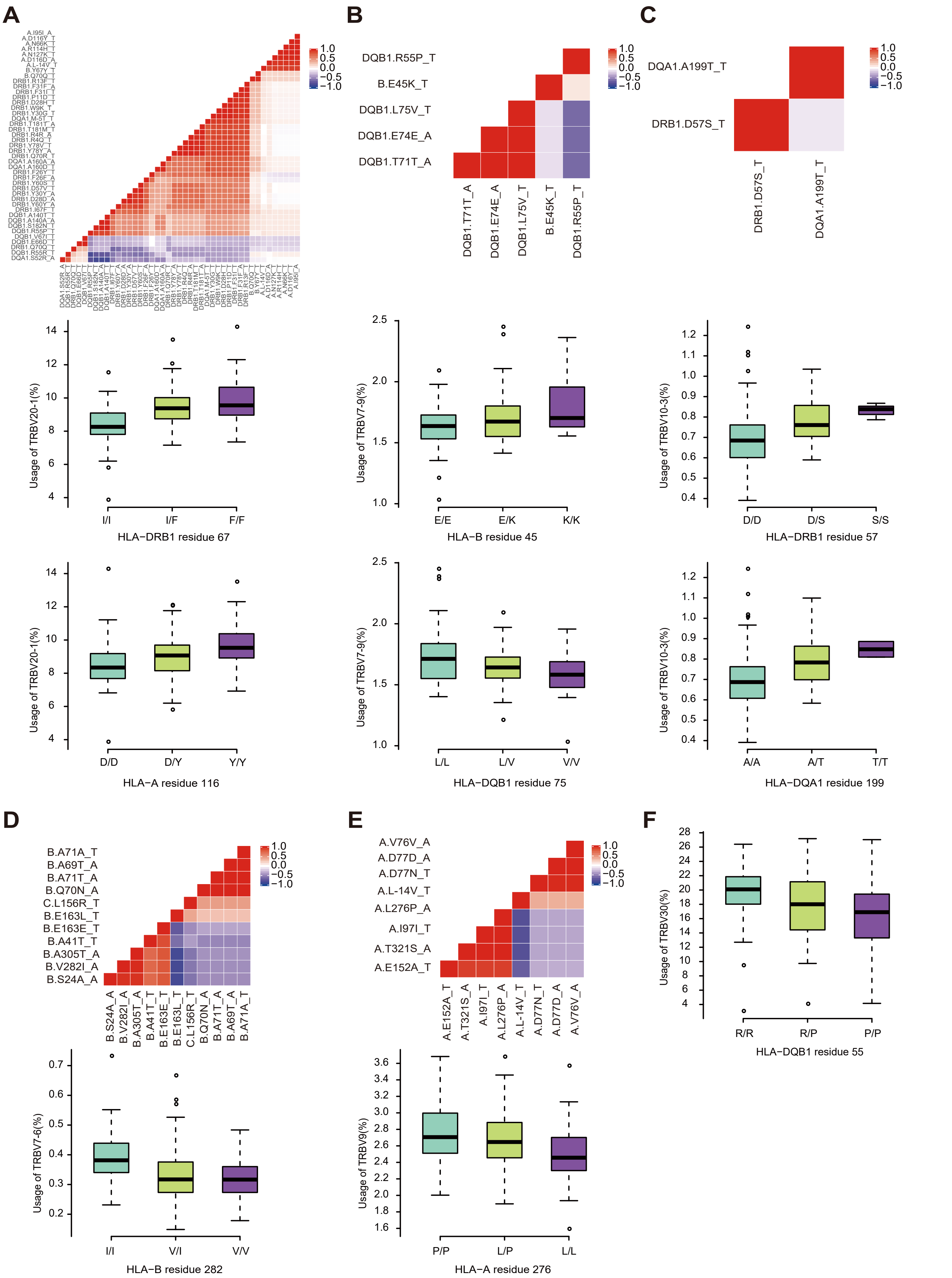

Supplementary Figure 2. Independent associations between other TRBV genes and amino acid variations in MHC alleles. Heat maps represent color-coded correlation matrix of the MHC amino acids that are significantly associated with the usage of TRBV20-1**(A)** TRBV7-9**(B)**, TRBV10-3**(C)**, TRBV7-6**(D)**, and TRBV9**(E)** (FDR ≤ 0.05), and the box plot in **(A-E)** show the independent associated amino acid variations with the specific TRBV gene. **(F)** The usage of TRBV30 gene has a single association with DQB1 residue 55. The TRBV gene usage is defined by the percentage of the number of sequences aligned to this V gene to the total number of sequences aligned to any V genes.
